# A29 ALBUMIN UTILISATION AT A TERTIARY CARE HOSPITAL

**DOI:** 10.1093/jcag/gwab049.028

**Published:** 2022-02-21

**Authors:** A Saad Mohammed, D Goldshtein, S Sarbar, S Wong, J Flemming

**Affiliations:** Queen’s University, Kingston, ON, Canada

## Abstract

**Background:**

Albumin is a colloidal solution with usage guided by recommendations from Canadian Blood Services (CBS). In Ontario, rates of albumin use increased substantially from 2012 to 2018 despite the lack of development of new indications for using over this time frame. Further, we recently found that >50% of albumin usage in patients with cirrhosis were non-evidence based. Albumin usage in other patient populations is not well described.

**Aims:**

Our aim was to describe overall usage of albumin stratified by clinical setting, clinical indication, and dosage at a tertiary care hospital.

**Methods:**

We retrospectively identified all albumin prescriptions during two randomly selected non-consecutive months between 2018 and 2019 at Kingston Health Sciences Centre. Data was abstracted from each hospital chart for indication, prescriber specialty, location of patient, amount of albumin ordered, and concentration (5% or 25%). Albumin prescriptions were then defined as either evidence-based or non-evidence based on published literature and guidelines. Overall cost for albumin during the study period was determined based on CBS pricing ($51.47/ 25gms).

**Results:**

A total of 699 albumin prescriptions were dispensed to 317 individuals over December 2018 and May 2019, with a total of 38,458 grams used. Overall, 36% was prescribed for evidence-based indications. The most common indication was plasmapheresis (32%), non-sepsis volume resuscitation (23%), and cardiac surgery (17%). The majority of albumin was prescribed in dialysis unit (32%), ICU (23%), and cardiac sciences unit (11%). The largest prescribers of albumin were intensivists (26%), followed by nephrologists (17%), and cardiac surgeons (15%). There were differences in utilization based on concentration of albumin. Despite limited evidence of benefit, 25% albumin and 5% albumin were used excessively for volume resuscitation and cardiac surgery respectively. When used for an evidence-based indication, the dosing was incorrect in 45% of orders. A total of $79,176 was spent on albumin during the study period. Importantly, only $28,494 was spent on evidence-based indications with appropriate dosage and concentration.

**Conclusions:**

Overall, there is significant albumin use for indications lacking substantial evidence. This study identifies the clinical contexts in which there is opportunity to reduce non-evidence-based albumin usage and cut unnecessary expenditure. Targeted quality improvement initiatives are underway.

**Funding Agencies:**

None

